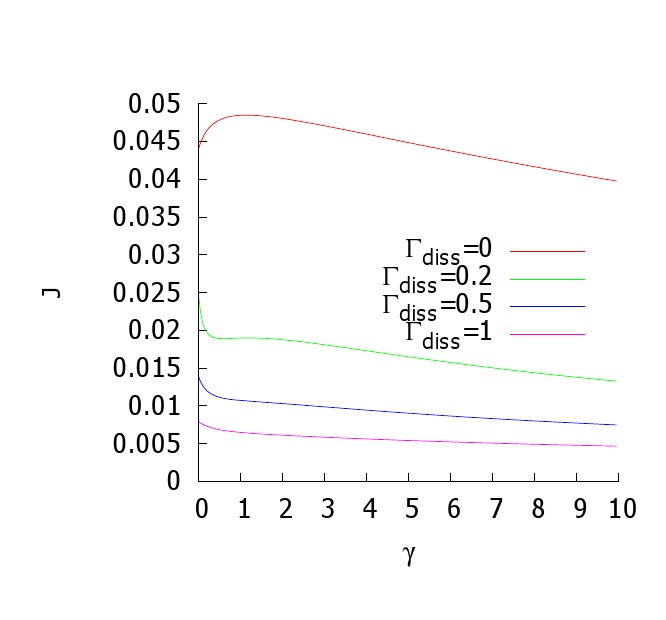# Correction: Quantum Transport in Networks and Photosynthetic Complexes at the Steady State

**DOI:** 10.1371/annotation/b73c06e9-1e54-497c-bafb-f56de94f2f18

**Published:** 2013-09-05

**Authors:** Daniel Manzano

There was an error in Figure 2. The right panel was a duplicate of the right panel in Figure 1.

The correct version of the right panel of Figure 2 is available here: 

**Figure:**